# ZGDHu-1 induces G_2_/M phase arrest and apoptosis in Kasumi-1 cells

**DOI:** 10.3892/mmr.2015.3160

**Published:** 2015-01-08

**Authors:** JUN XIA, SU-FENG CHEN, YA-PING LV, LING-NA LU, WEI-XIAO HU, YONG-LIE ZHOU

**Affiliations:** 1 Centre of Laboratory Medicine, Zhejiang Provincial People’s Hospital, Hangzhou, Zhejiang 310014, P.R. China; 2 College of Medical Technology, Zhejiang Chinese Medical University, Hangzhou, Zhejiang 310053, P.R. China; 3 College of Pharmaceutical Science, Zhejiang University of Technology, Hangzhou, Zhejiang 310014, P.R. China; 4 School of Laboratory Medicine and Life Science, Wenzhou Medical University, Wenzhou, Zhejiang 325035, P.R. China

**Keywords:** Kasumi-1, N,N′-di-(m-methylphenyi)-3, 6-dimethyl-1,4-dihydro-1,2,4,5-tetrazine-1,4-dicarboamide, cell cycle arrest, checkpoint kinase 1

## Abstract

The present study examined the effects of N,N′-di-(m-methylphenyi)-3, 6-dimethyl-1, 4-dihydro-1,2,4,5-tetrazine-1,4-dicarboamide (ZGDHu-1), a novel oxazine derivative, in Kasumi-1 cells. Following incubation with various concentrations of ZGDHu-1, fluorescence-activated cell sorting (FACS) was used in order to detect changes in mitochondrial membrane permeability in Kasumi-1 cells. Western blot analysis was performed in order to analyze the expression of nuclear factor-κB, inhibitor of κB and AML1/ETO. In addition FACS was used to analyze leukemia cell cycles and the expression levels of cyclin, cyclin-dependent kinases and cyclin-dependent kinase inhibitors in G_2_/M phase were determined using FACS and western blot analysis. The upregulation of reactive oxygen species production and mitochondrial membrane permeability was ascribed to apoptosis. The growth of Kasumi-1 cells was inhibited through the downregulation of nuclear factor-κB, degradation of AML1/ETO fusion protein and cell cycle arrest at the G_2_/M phase. This study documented that G_2_/M regulatory molecules, including cyclin B1, cell division control (cdc)2 and cdc25c were downregulated and checkpoint kinase 1 (CHK1), p53, p27, phospho-cdc25c, phospho-CHK1 and phospho-p53 were upregulated following treatment with ZGDHu-1. In the present study, pretreatment with CHIR-124, a selective CHK1 inhibitor, abrogated G_2_/M arrest via ZGDHu-1. These results demonstrated the anti-tumor activity of ZGDHu-1, which may therefore a potential target for further investigation and may be useful for the treatment of patients with t(8;21) acute myeloid leukemia.

## Introduction

Leukemia is a type of malignant cancer of the hematopoietic stem cells and is a severe threat to human health. At present, the main therapy for leukemia includes chemotherapy, allogeneic hematopoietic stem cell transplantation, bone marrow transplantation, targeted drug therapy and immunotherapy.

Genetic abnormalities have been demonstrated to be important in leukemogenesis (1). The AML1/ETO (A/E) fusion gene is a product of the chromosome translocation t(8;21) (q22; q22), which affects the acute myeloid leukemia gene 1 (AML1) of chromosome 21 and the eight twenty one gene (ETO) of chromosome 8. The fusion gene is one of the common chromosome aberrations in acute myeloid leukemia (AML), particularly in AML with maturation (M2) (2).

Aggressive cytosine arabinoside-based chemotherapy is the standard protocol for the treatment of t(8;21) AML. However, clinical observation has demonstrated that the median survival time of patients with t(8;21) AML was >2 years, with a 5 year survival rate of <40% (2). The development of novel therapies is required in order to further improve clinical outcome and to provide therapeutic options for t(8;21) AML patients.

N,N′-di-(m-methylphenyi)-3, 6-dimethyl-1, 4-dihydro-1,2,4,5-tetrazine-1,4-dicarboamide (ZGDHu-1) is a novel oxazine derivative synthesized by Professor Wei-Xiao Hu from the College of Pharmaceutical Science, Zhejiang University of Technology (Hangzhou, China) who obtained a Patent of China (3,4). ZGDHu-1 has been demonstrated to possess anti-tumor activity (5,6) and has also been found to inhibit growth and induce apoptosis of Kasumi-1 cells (7), a cell line of the t(8;21) (q22;q22) translocation. ZGDHu-1 is also able to markedly inhibit the cell cycle at the G_2_/M phase, however, the underlying mechanisms were not discussed.

The cell cycle includes the mitotic period (M), the G_1_ phase, the S phase and the G_2_ phase. Its regulation predominantly depends on the regulatory network, including cyclins, cyclin-dependent kinases (CDKs) and cyclin-dependent kinase inhibitors (CKIs) (8,9). The checkpoint of G_2_/M is important for entrance of cells into the M phase and is also important in tumor cell resistance (10). When the cell cycle arrests at the G_2_/M phase, the expression levels of the CDK1/cyclin B1 complex are altered, leading to incomplete mitosis and ultimately mitotic catastrophe resulting in cell death (11).

Thus, in the present study, we aimed to investigate the mechanism by which ZGDHu-1 induces G2/M phase arrest and apoptosis in Kasumi-1 cells.

## Materials and methods

### ZGDHu-1

ZGDHu-1 was kindly provided by the Pharmaceutical Engineering Research Institute, College of Pharmaceutical Science, Zhejiang University of Technology. It was screened from 14 compounds of 3,6-disubstituted-1,4-dihydro-1,2,4,5-tetrazine derivatives. Stock solution (10 mg/ml) was prepared by dissolving ZGDHU-1 in dimethyl sulfoxide (DMSO; Simga-Aldrich, St. Louis, MO, USA), then aliquoted and stored at -20°C. For the *in vitro* experiment, RPMI-1640 medium (Gibco, Grand Island, NY, USA) was used to prepare the final working concentration.

### Cell culture and drug treatment

Kasumi-1 cells (12), derived from the peripheral blood of a 7 year old Japanese male who was diagnosed with AML-M2, were purchased from the American Type Culture Collection (Manassas, VA, USA) and were maintained in RPMI-1640 with 20% fetal bovine serum (Gibco). The genetic characteristics of this cell line include a chromosome t(8;21) (q22;q22) translocation, thus making it a good research tool for investigating this type of translocation in leukemia. The cell line was incubated in a 37°C humidified atmosphere with 5% CO_2_. Different concentrations of ZGDHu-1 (50, 100, 200, 500 and 1,000 μg/l) and controls (negative control and DMSO as solvent control) were added to the Kasumi-1 cells.

### Flow cytometric analysis

DNA Prep™ reagent system (Beckman Coulter, Indianapolis, IN, USA) was used to evaluate cell cycle alterations in Kasumi-1 cells. The cells were harvested following washing with phosphate-buffered saline (PBS; DingGuo Biotechnology Co., Ltd., Beijing, China). DNA Prep LPR (50 μl; Beckman Coulter) was added for 1 min and then 150 μl DNA Prep stain was added to the cells. Following gentle agitation, the cells were incubated for 5 min at room temperature, the results were detected using fluorescence-activated cell sorting (FACS) using a Coulter Epics XL flow cytometer (Beckman Coulter) and the proportion of cells at each stage of the cell cycle was determined.

To elucidate the underlying mechanisms of apoptosis, the expression of certain apoptosis-associated proteins were analyzed using FACS. To detect Apo 2.7, collected cells were permeabilized for 20 min at 4°C with 100 μg/ml digitonin and then phycoerythrin-labeled Apo 2.7 mouse monoclonal (mAb) immunoglobulin G antibody (IM2088U; Beckman Coulter) was added for 15 min.

Collected cells were stained with propidium iodide (PI, 10 μg/ml) and rhodamine 123 (Rh123, 10 μg/ml; Calbiochem, San Diego, CA, USA) to detect the mitochondrial transmembrane potentials using FACS analysis.

Dihydrorhodamine 123 (DHR123; Sigma-Aldrich) was used to detect the ROS levels of collected cells (13). Following being washed in PBS, 150 μl 10 μM DHR123 was added to the cells. Subsequently, the cells were incubated at 37°C for 30 min. FACS was used to measure the changes in median fluorescence intensity (MFI).

IntraPrep permeabilization reagent (Beckman Coulter) was used to detect the expression of the following intracellular proteins: B-cell lymphoma 2 (Bcl-2), Bcl-2-associated death promoter (Bad), Bcl-2-associated X protein (Bax) and cyclin B1. A total of 50 μl fixation reagent was added to the collected cells and then incubated for 15 min at room temperature. Following being washed with PBS, 50 μl permeabilization reagent was added. After 5 min incubation, specific antibodies, including Bcl-2 (BD Biosciences, San Jose, CA, USA), Bad (Biovision, Mountain View, CA, USA), Bax (BD Biosciences) and mouse mAb cyclin B1 (1:2,000; #4135; Cell Signaling Technology, Inc., Beverly, MA, USA) were added to the cells. The cells were incubated for 15 min in the dark at room temperature and then FACS was used to determine the results.

### Western blot analysis

Following incubation with different concentrations of ZGDHU-1, Kasumi-1 cells were lysed and proteins were extracted and quantitated using a bicinchoninic protein assay kit (DingGuo Biotechnology Co., Ltd.). The proteins were loaded into wells of an 8 or 12% SDS-PAGE, electrophoresed and transferred onto a nitrocellulose membrane (DingGuo Biotechnology Co., Ltd.). The membrane was incubated with the appropriate primary antibody and then washed and incubated with horseradish peroxidase-conjugated secondary antibody (Cell Signaling Technology, Inc.). Detection was performed using a western blotting luminol reagent (Santa Cruz Biotechnology, Inc., Dallas, TX, USA; cat no. sc-2048). The following antibodies were used: Rabbit mAb caspase-3 (1:1,000; #9665), rabbit polyclonal (pAb) cleaved caspase-3 (1:1,000; #9661), rabbit mAb poly ADP ribose polymerase (PARP; 1:1,000; #9532), rabbit pAb NF-κB (1:1,000; #3034), rabbit pAb inhibitor of κB (IκB)-α (1:1,000; #9242), rabbit pAb Bax (1:1,000; #2774), rabbit mAb Bad (1:1,000; #9239), rabbit mAb AML1 (1:1,000; #4336), rabbit pAb ETO (1:1,000; #4498), mouse mAb cyclin B1 (1:2,000; #4135), mouse mAb cdc2 (1:2,000; #9116), rabbit mAb cdc25C (1:1,000; #4688), rabbit mAb phospho-cdc25C (Ser216) (1:1,000; #4901), rabbit pAB Chk1 (1:1,000; #2345), rabbit mAb phospho-Chk1 (Ser345) (1:1,000; #2348), mouse mAb Chk2 (1:1,000; #3440), mouse mAb phospho-p53 (Ser15)(1;1,000; #9286), rabbit pAb phospho-p53 (Ser20) (1:1,000; #9287), rabbit mAb p27 (1:1,000;#3688), which were all purchased from Cell Signaling Technology, Inc., as well as mouse mAb Bcl-2 (1:250; sc-7382), mouse mAb β-actin (1:1,000; sc-47778) and rabbit pAb p53 (1:1,000; sc-6243), which were purchased from Santa Cruz Biotechnology, Inc.

### Treatment with CHIR-124, a selective CHK1 inhibitor

In a separate experiment, 200 nM CHIR-124 (14,15), a selective CHK1 inhibitor, was incubated with Kasumi-1 cells for 2 h and then 200 μg/l ZDGHU-1 for 48 h. Cell cycle stages were analyzed by FACS to observe differences.

### Statistical analysis

All statistical calculations were performed using SPSS 15.0 software (SPSS, Inc., Chicago, IL, USA). Results are expressed as the mean ± standard deviation. The differences between treated and control groups were analyzed using a one-way analysis of variance or χ^2^ test. P<0.05 was considered to indicate a statistically significant difference.

## Results

### ZGDHu-1 induces Kasumi-1 cell apoptosis through the activation of caspase-3 and the mitochondrial apoptosis pathway

**Figure 1:**
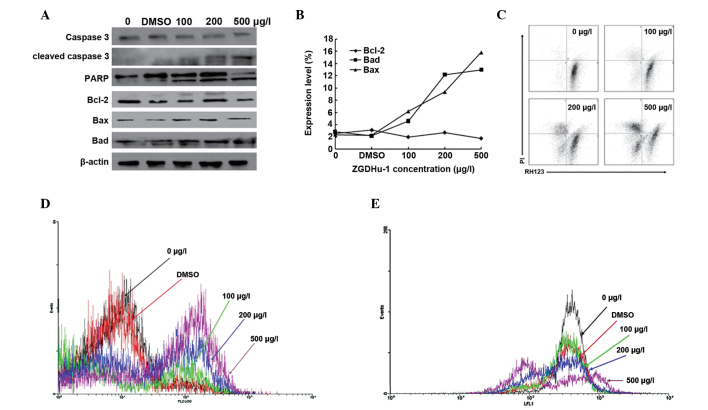
ZGDHu-1 induces Kasumi-1 cell apoptosis through the activation of caspase-3 and mitochondrial apoptosis pathway (A) Effect of ZGDHu-1 on caspase-3, cleaved caspase-3, PARP, Bcl-2, Bax and Bad. There was a negative control with no drugs and a DMSO control. β-actin was used as a loading control. (B) Expression levels of Bcl-2, Bax and Bad were detected by fluorescence activated cell sorting. (C) Kasumi-1 cells stained with propidium iodide and Rh123 were detected by fluorescence activated cell sorting. (D) Results of Apo 2.7 following treatment with ZGDHU-1 in Kasumi-1 cells. Black indicates the negative control, red indicates the DMSO control, green indicates 100 μg/l ZGDHU-1, blue indicates 200 μg/l ZGDHU-1 and brown indicates 500 μg/l ZGDHU-1. (E) ROS production was induced in Kasumi-1 cells. Rh123, the product of DHR123 and ROS, was measured by flow cytometry. Black indicates the negative control, red indicates the DMSO control, green indicates 100 μg/l ZGDHU-1, blue indicates 200 μg/l ZGDHU-1 and brown indicates 500 μg/l ZGDHU-1. ZGDHu-1, N,N′-di-(m-methylphenyi)-3,6-dimethyl-1,4-dihydro-1,2,4,5-tetrazine-1,4-dicarboamide; Bcl-2, B-cell lymphoma 2; Bax, Bcl-2-associated X protein; Bad, Bcl-2-associated death promoter; ROS, reactive oxygen species; DMSO, dimethyl sulfoxide; PARP, poly ADP ribose polymerase; Rh123, rhodamine 123.

The caspase family is important in the apoptotic signaling pathway (16). In the present study, alterations in caspase-3, cleaved caspase-3 (the active part of caspase-3) and PARP (the substrate of caspase-3) were assessed following treatment with different concentrations of ZGDHu-1 for 48 h in Kasumi-1 cells using western blotting. Caspase-3 decreased and cleaved caspase-3 markedly increased in a dose-dependent manner. The cleaved fragments of PARP (89 kDa) were also easily observed, which suggested that caspase-3 was activated following ZGDHu-1 treatment (Fig. 1A).

FACS and western blotting were used to detect alterations in the expression of Bcl-2, Bad and Bax following treatment with different concentrations of ZGDHu-1 for 48 h in Kasumi-1 cells. ZGDHu-1 treatment led to an upregulation of Bad and Bax, however, Bcl-2 was not altered (Fig. 1A and B)

In order to evaluate the effects on the mitochondrial signaling pathway, FACS was used to detect alterations in mitochondrial membrane protein (Apo 2.7) (17) and Δψm, which reflects the integrity of the mitochondrial membrane. The Kasumi-1 cells were double-stained with PI and Rh123 (18), which was proportional to Δψm. Following treatment with different concentrations of ZGDHu-1 (control, DMSO control, 100, 200 and 500 μg/l), the MFI was significantly decreased between 40.4±1.6, 39.1±2.2, 33.3±2.6, 30.3±2.4 and 27.7±1.9 (P<0.05) in PI negative and Rh123 positive cells (Fig. 1C). In addition, the expression of Apo 2.7 was increased significantly between 5.35±0.4, 5.30±0.3, 9.73±1.4, 36.90±1.6 and 54.40±1.8% (P<0.05; Fig. 1D).

### ZGDHu-1 induces ROS production in Kasumi-1 cells

Overproduction of ROS may cause oxidative stress, which is a major factor leading to apoptosis. In order to investigate whether ROS accumulation had occurred, DHR123, one of the most widely used ROS probes for intracellular measurement and analysis, was used. DHR123 is not fluorescent until oxidized by ROS to the highly fluorescent product rhodamine 123, therefore, MFI was measured using flow cytometry. The MFI was increased to 26.6±1.2, 25±1.4, 24.2±1.0, 23.7±1.6 and 38.3±2.1 (P<0.05) at a concentration of 500 μg/l (Fig. 1E).

### ZGDHu-1 downregulates the expression of NF-κB and upregulates the expression of IκB

**Figure 2:**
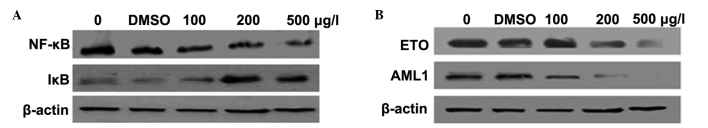
ZGDHu-1 downregulates the expression of NF-κB and upregulates the expression of IκB, causing degradation of the A/E fusion protein (A) Effect of ZGDHu-1 on NF-κB and IκB. There was a negative control with no drugs and a DMSO control. β-actin was used as a loading control. (B) Protein levels of the A/E fusion gene following treatment with ZGDHu-1. Western blot analysis was used to detect changes in these genes. β-actin was used as a loading control. ZGDHu-1, N,N′-di-(m-methylphenyi)-3,6-dimethyl-1,4-dihydro-1,2,4,5-tetrazine-1,4-dicarboamide; NF-κB, nuclear factor-κB; DMSO, dimethyl sulfoxide; AE, AML1/ETO.

Following being incubated with different concentrations of ZGDHu-1 (0, 100, 200 and 500 μg/l), the expression levels of NF-κB and IκB were detected using western blotting. The result suggested that IκB levels were induced by ZGDHu-1 to suppress the function of NF-κB and inhibit the growth of Kasumi-1 cells (Fig. 2A).

### Effect of ZGDHu-1 on the A/E fusion gene of Kasumi-1 cells at the mRNA and protein level

The A/E fusion gene is a vital feature of Kasumi-1 cells and it also has a major role in the development of AML. Therefore, in the present study, alterations in the expression of this fusion gene following treatment with different concentrations of ZGDHu-1 were investigated. The result demonstrated that A/E fusion levels did not change at the mRNA level (data not shown), but AML and ETO genes were degraded by ZGDHU-1 (Fig. 2B).

### ZGDHu-1 induces G_2_/M arrest in Kasumi-1 cells

**Figure 3:**
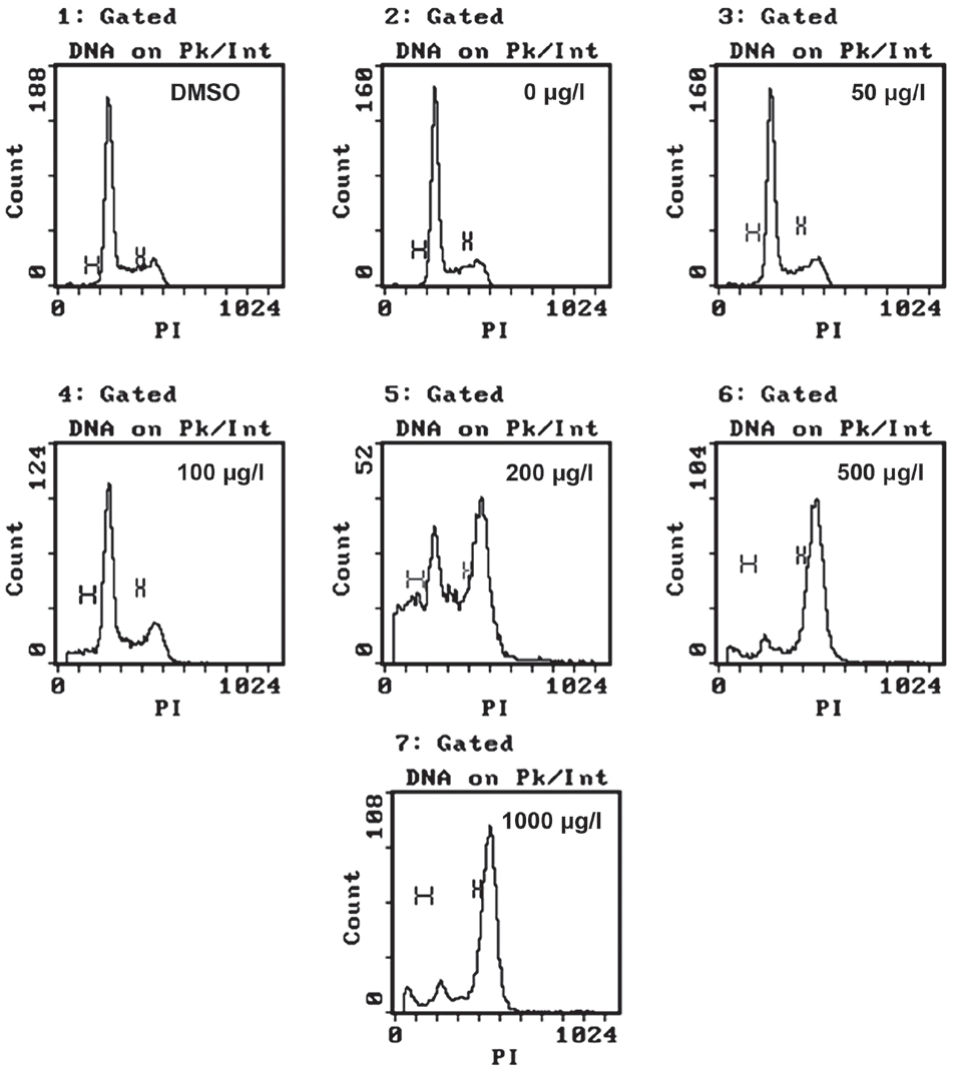
Cell cycle effects of Kasumi-1 cells treated with ZGDHu-1. Fluorescence activated cell sorting was used to detect cell cycle alterations in Kasumi-1 cells. The phenomena of G_2_/M arrest was observed. ZGDHu-1, N,N′-di-(m-methylphenyi)-3,6-dimethyl-1,4-dihydro-1,2,4,5-tetrazine-1,4-dicarboamide; PI, propidium iodide.

Following incubation with different concentrations of ZGDHu-1 (50, 100, 200, 500 and 1,000 μg/l) and controls (negative control and DMSO control) for 48 h, cell cycle stages were analyzed using FACS (Fig. 3). The present results demonstrated that cells in the G_2_/M phase significantly accumulated in a concentration-dependent manner (between 6.4±1.5, 13.4±1.3, 40.1±1.4, 81.2±1.4 and 79.9±1.4%). Based on this observation, it was hypothesized that the inhibition and apoptotic effects of Kasumi-1 cells may operate through the disturbance of the cell cycle check point.

### Activation of CHK1 and p53 induces G_2_/M arrest in Kasumi-1 cells

To further investigate the molecular mechanism for ZGDHu-1-induced G_2_/M arrest in Kasumi-1 cells, the protein levels of cyclin B1 were analyzed using FACS. Additionally, certain CDKs and CKIs were measured using western blotting.

**Figure 4:**
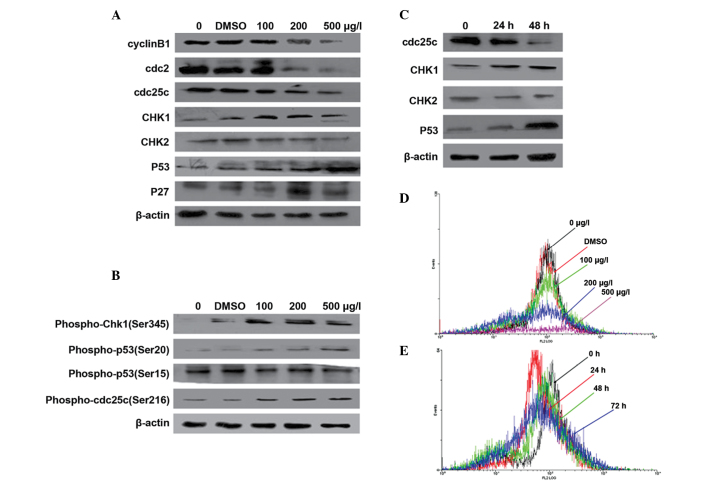
Expression changes in G_2_/M-associated CDKs and CKIs. (A) Western blot analysis of the level of G_2_/M cell cycle control protein. Cyclin B1, cdc2, cdc25c, CHK1, CHK2, p53 and p27 were treated with different concentrations of ZGDHU-1 and controls for 48 h. β-actin was used as a loading control. (B) Western blot analysis of the level of phospho-cdc25c at Ser216, phospho-Chk1 at Ser345, phospho-p53 at Ser20 and Ser15. (C) Western blot analysis of cdc25c, CHK1, CHK2, p53 at 200 μg/l ZGDHU-1 with different cultivating times. (D) Expression of cycin B1 with different concentrations of ZGDHU-1 and controls for 48 h using fluorescence activated cell sorting. The black line indicates the negative control, red line indicates the DMSO control, green line indicates 100 μg/l ZGDHU-1, blue line indicates 200 μg/l ZGDHU-1 and the brown line indicates 500 μg/l ZGDHU-1. (E) Expression of cycin B1 at 200 μg/l ZGDHU-1 with different cultivating times using fluorescence activated cell sorting. Black indicates 0 h, red indicates 24 h, green indicates 48 h and blue indicates 72 h. ZGDHu-1, N,N′-di-(m-methylphenyi)-3,6-dimethyl-1,4-dihydro-1,2,4,5-tetrazine-1,4-dicarboamide; CDK, cyclin-dependent kinase; CKI, cyclin-dependent kinase inhibitor; cdc, cell division ccontrol; DMSO, dimethyl sulfoxide; CHK, checkpoint kinase.

The combination of cyclin B1 and cdc2 is an important step for eukaryotic cells entering into mitosis (19). The present result implied that the protein level of cdc2 (Fig. 4A) and cyclin B1 (Fig. 4A, D and E) were markedly decreased in a concentration- and time-dependent manner, which inhibited the number of Kasumi-1 cells entering mitosis.

There are also additional CDKs and CKIs, which have important roles in G_2_/M arrest. Cdc25c is a protein phosphatase responsible for dephosphorylating and activating cdc2, while phosphorylation at Ser216 is DNA damage dependent at the G_2_/M checkpoint. Therefore, the expression of cdc25c and phospho-cdc25c at Ser216 was detected and the result revealed that the level of cdc25c was decreased in a concentration- and time-dependent manner, while phospho-cdc25c was increased in a concentration-dependent manner (Fig. 4A–C).

CHK1 and CHK2 are important in DNA damage check point control (20,21), therefore, their protein levels were evaluated in order to determine through which pathway ZGDHu-1 induced G_2_/M arrest in Kasumi-1 cells. The expression of CHK1 was significantly increased in a concentration- and time-dependent manner, while no difference was identified in the expression of CHK2 and phospho-CHK1 (Ser245), which exhibited similar results compared with CHK1 (Fig. 4A–C).

p53, a well-known tumor suppressor, has various roles in the cellular response to damage information (22). In the present study, p53 was activated in a time- and concentration-dependent manner when treated with ZGDHu-1 (Fig. 4A–C). Phospho-p53 at Ser20 site was also upregulated via CHK1, while no difference was identified at the Ser15 site (Fig. 4B). P27, a CKI, was also upregulated (Fig. 4A).

### CHIR-124 reduces the proportion of cells in the G_2_/M phase and alters the expression of certain CDKs

**Figure 5:**
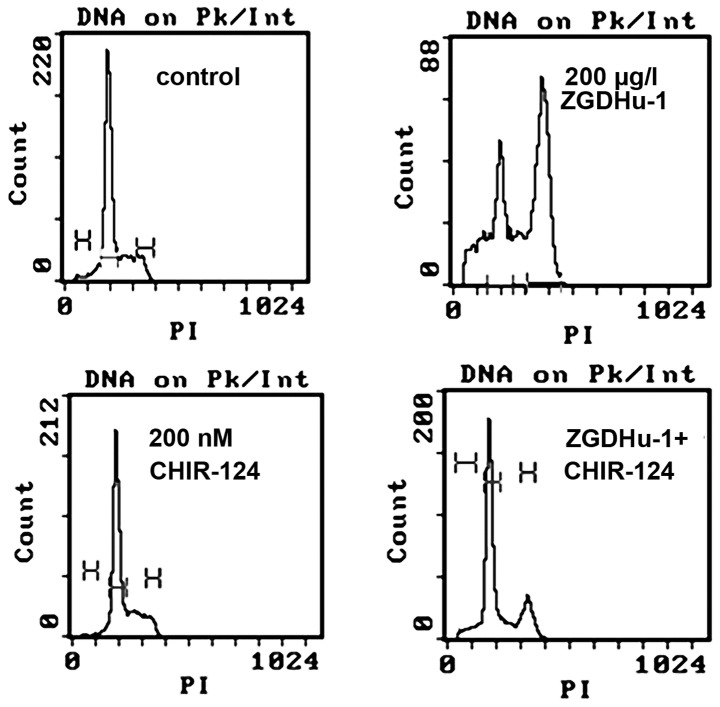
Effects of the CHK1 inhibitor on ZGDHu-1-treated Kasumi-1 cells. The CHIR-124 inhibitor reduced cell cycle arrest in ZGDHu-1-treated cells. Fluorescence-activated cell sorting was used to analyze the cell cycle of Kasumi-1 cells, which were treated with ZGDHu-1 or CHIR-124 alone or with both. ZGDHu-1, N,N′-di-(m-methylphenyi)-3,6-dimethyl-1,4-dihydro-1,2,4,5-tetrazine-1,4-dicarboamide; CHK, checkpoint kinase; PI, propidium iodide.

To confirm the role of CHK1 in ZGDHu-1-mediated G_2_/M arrest, Kasumi-1 cells were treated with 200 nM of the CHK1 inhibitor prior to ZGDHu-1 treatment. As shown in Fig. 5, the proportion of cells in the G_2_/M phase reduced from 40.1±1.4 to 8.6±1.2% (P<0.05), while cells in the G_0/1_ phase increased.

## Discussion

Our previous study revealed that ZGDHu-1 may inhibit the growth of Kasumi-1 cells in a time- and dose-dependent manner *in vitro*, however, the underlying mechanisms have not been discussed. In the present study, ZGDHu-1 induced apoptosis through the activation of caspase-3. Caspases are a family of cytosolic aspartate-specific cysteine proteases, which are involved in the initiation and execution of apoptosis. They are expressed as latent zymogens and are activated by an autoproteolytic mechanism or by processing by other proteases. Within the caspase family, caspase-3 is a key enzyme and it was demonstrated to be activated following treatment with ZGDHu-1 in leukemia cells (23). Overall, the present results demonstrate that ZGDHu-1 may induce Kasumi-1 cell apoptosis. Furthermore, ZGDHu-1 may arrest the cell cycle at the G_2_/M phase of leukemia cells when the concentration of ZGDHu-1 was 100 μg/l.

NF-κB is a protein complex that controls the transcription of DNA, while IκB is an inhibitory factor (24); activation of the NF-κB signaling pathway is initiated by the signal-induced degradation of IκB proteins. With the degradation of IκB, the NF-κB complex is then translocated to the nucleus where it can ‘turn on’ the expression of specific genes that have DNA-binding sites for NF-κB. Furthermore, the present data revealed that ZGDHu-1 may upregulate the expression of IκB and downregulate the expression of NF-κB to inhibit the growth of Kasumi-1 cells. The A/E fusion gene is a vital characteristic of Kasumi-1 cells and it also has a major role in the biology of this type of leukemia. In the present study, it was revealed that ZGDHu-1 may significantly decrease the protein level of this fusion gene, suggesting that ZGDHU-1 may effectively inhibit the development of this leukemia partly through this mechanism.

There are two major pathways able to induce apoptosis (25), which are classified as the extracellular (extrinsic inducers) or intracellular (intrinsic inducers) pathway. Mitochondrial regulation is a vital part of the intracellular pathway. The changes in mitochondrial membrane protein (Apo 2.7) and Δψm suggested that the integrity of the mitochondrial membrane was destroyed. There was also ROS accumulation in the Kasumi-1 cells. The changes in Bcl-2, Bad and Bax determined via FACS and western blotting confirmed that ZGDHu-1 induced apoptosis through the mitochondrial pathway (26).

The cell cycle is a series of events that take place in a cell leading to its division and duplication. It includes the mitotic period and interphase; interphase may be further subdivided into three phases, which include the G_1_ phase, S phase and G_2_ phase. In the present study, ZGDHu-1 induced G_2_/M phase arrest in Kasumi-1 cells in a concentration-dependent manner.

Numerous proteins and kinases are involved in the process of the cell cycle. The protein levels of cdc2 and cyclin B1 were markedly decreased in a concentration-dependent manner, leading to the obstruction of mitotic entry in Kasumi-1 cells. The level of cdc25c was also decreased; cdc25c is a protein phosphatase responsible for dephosphorylating and activating cdc2, while phosphorylation at Ser216 is DNA damage dependent at the G_2_/M checkpoint. The present results demonstrated that phospho-cdc25c was increased to inhibit the combination of cdc2 and cyclin B1. p53 was activated and p27, as CKIs, were upregulated to induce G_2_/M arrest.

In order to elucidate whether CHK1 is important in ZGDHu-1-induced cell cycle arrest in Kasumi-1 cells, western blotting was used to detect the protein level of CHK1 and p-CHK1, which revealed an increased concentration of this protein. In addition, a type of CHK1 inhibitor was added prior to ZGDHu-1 administration. Following being incubated with ZGDHu-1, it was revealed that the proportion of cells in the G_2_/M reduced, while the number of cells in the G_0/1_ phase increased.

In conclusion, the present study demonstrated that ZGDHu-1 was able to inhibit the proliferation and induce the apoptosis of Kasumi-1 cells. Notably, this compound was able to arrest the cell cycle at the G_2_/M phase. CHK1 kinase was found to be important in these activities. The present results suggested that ZGDHu-1 may be a potential drug to treat leukemia in the future.
